# Mutations in the PH Domain of *DNM1* are associated with a nonepileptic phenotype characterized by developmental delay and neurobehavioral abnormalities

**DOI:** 10.1002/mgg3.362

**Published:** 2018-02-04

**Authors:** Emily Brereton, Emily Fassi, Gabriel C. Araujo, Jonathan Dodd, Aida Telegrafi, Sheel J. Pathak, Marwan Shinawi

**Affiliations:** ^1^ Washington University School of Medicine St. Louis MO USA; ^2^ Division of Genetics and Genomic Medicine Department of Pediatrics Washington University School of Medicine St. Louis MO USA; ^3^ Department of Psychology St Louis Children's Hospital St. Louis MO USA; ^4^ GeneDx Gaithersburg MD USA; ^5^ Department of Neurology Washington University School of Medicine St. Louis MO USA

**Keywords:** developmental delay, domain, dynamin 1, epileptic encephalopathy, intellectual disability, synaptic vesicle

## Abstract

**Background:**

Dynamin 1 is a protein involved in the synaptic vesicle cycle, which facilitates the exocytosis of neurotransmitters necessary for normal signaling and development in the central nervous system. Pathogenic variants in *DNM1* have been implicated in global developmental delay (DD), severe intellectual disability (ID), and notably, epileptic encephalopathy. All previously reported *DNM1* pathogenic variants causing this severe phenotype occur in the GTPase and Middle domains of the dynamin 1 protein.

**Methods:**

We used whole‐exome sequencing to characterize the molecular basis of DD and autistic symptoms in two identical siblings.

**Results:**

The twin siblings exhibit mild to moderate ID and autistic symptoms but no epileptic encephalopathy. Exome sequencing revealed a genetic variant, c.1603A>G (p.Lys535Glu), in the PH domain of dynamin 1. Previous in vitro studies showed that mutations at Lys535 inhibit endocytosis and impair PH loop binding to PIP2.

**Conclusions:**

Our data suggest a previously undescribed milder phenotype associated with a missense genetic variant in the PH domain of dynamin 1.

## 1 BACKGROUND

Neurotransmission in the central nervous system depends on synaptic vesicle transport. Neurotransmitters are taken up by exocytotic vesicles which fuse with the plasma membrane, releasing neurotransmitters into the synapse (Sudhof, 2004). Neurotransmitters in the synapse are degraded or taken up into the presynaptic axon. Vesicle endocytosis, when vesicles bud off from the plasma membrane into the presynaptic axon, is mediated by proteins including amphysin, endophilin, clathrin, and dynamin (Rizzoli, 2014). Newly synthesized or retrieved neurotransmitters enter these new vesicles and restart the cycle (Takei, 1996).

The Dynamin family of proteins interact with clathrin in the vesicle budding cycle and participate in the scission between the cell membrane and vesicle wall. After the invagination of the lipid membrane, dynamin binds GTP and its alpha helixes wrap around and pinch the budding vesicle. Next, dynamin uses the energy from GTP hydrolysis to sever the membrane tubules and complete vesicle fission (Antonny et al., 2016). This process is critical for the synaptic vesicle cycle (McNiven, 2000). Dynamin proteins have five domains; the GTPase domain is the largest and best understood, followed by a middle domain, pleckstrin homology (PH) domain, coiled coil (CC) domain, and a proline‐rich domain (PRD) (McNiven, 2000). The PH domain is thought to interact directly with the lipid bilayer (Vallis, Wigge, Marks, Evans, & McMahon, 1999).

The *DNM1* gene (MIM 602377) codes for dynamin 1 and is mainly expressed in the central nervous system (Romeu & Arola, 2014). Pathogenic *DNM1* variants affect brain development and function and cause epileptic encephalopathy associated with global developmental delay (DD) (Allen et al., 2016; Deng et al., 2016; Epi4K Consortium; Epilepsy Phenome/Genome Project, 2013; Euro E‐RESC, EpilepsyPhenome/Genome P, Epi4K Constorium, 2014; Nakashima et al., 2016). Previously reported patients with *DNM1*‐related disorder carry pathogenic variants in the GTPase or middle domains, but never in the PH domain (Allen et al., 2016; Deng et al., 2016; Epi4K Consortium; Epilepsy Phenome/Genome Project, 2013; Euro E‐RESC, EpilepsyPhenome/Genome P, Epi4K Constorium, 2014; Nakashima et al., 2016).

Here, we report 8‐year‐old identical twin sisters with a de novo p.Lys535Glu likely pathogenic novel variant in exon 15 of *DNM1* (NM_004408.3), the first documented pathogenic variant in the PH domain. Although all previously reported patients with *DNM1* pathogenic variants exhibited epileptic encephalopathy and severe neurological phenotype, the two probands in this report had no seizures and exhibited mild to moderate DD/intellectual disability (ID) and autism spectrum disorder. Our data suggest an emerging and previously undescribed phenotype associated with a specific *DNM1* missense genetic variant in the PH domain.

## 2 CLINICAL REPORT

Patients 1 and 2 are monozygotic triplet sisters who presented for evaluation of DD, autism spectrum disorder, some dysmorphic features, and hypotonia. They were born at 36 weeks gestation, which was conceived naturally, via an uncomplicated cesarian section secondary to triplet pregnancy. There were no complications after delivery. They have a healthy triplet brother. Patient 1 had a birth weight of 2.8 kg (>97th percentile for triplet gestation), a birth length of 49 cm (>97th percentile), and an occipitofrontal circumference (OFC) of 33.8 cm (80th percentile). Patient 2 had a birth weight of 2.55 kg (50th–90th percentile for triplet gestation), a birth length of 48.2 cm (>97th centile), and OFC of 33.1 cm (65th percentile). Their triplet brother had a birth weight of 2.38 kg (50th–90th percentile for triplet gestation), a birth length of 46.9 cm (97th percentile), and OFC of 32 cm (40th percentile).

Developmental concerns were first noted at 4 months of age when both sisters were noted to lack visual tracking, but this spontaneously resolved at 7 months of age. Patient 1 and 2 started receiving physical, occupational, and speech therapies at 1 year of age due to delayed speech and motor milestones. Both sisters started sitting at 7–8 months, crawling at 11 months, and walking at 18 months. At 18 months, Patient 1 could say 5–10 words, but lost most of this vocabulary by age 2. Patient 2 had delayed language acquisition but no language loss. Their triplet brother started sitting at 6 months, crawling at 9 months and walking at 15 months. He required speech therapy for 9 months during third year of life for mild speech delay. The brother is in mainstream classes, never required any resources at school, and does not exhibit neurobehavioral abnormalities. The sisters were diagnosed with autism at 3 years of age and have been receiving applied behavior analysis (ABA) therapy since. At the age of 5, Patient 2 had one episode of febrile seizure. She had a normal brain MRI after this event. She also had a few staring episodes, but her EEG at 7 years of age was normal.

Developmental evaluation of both sisters at age 8 years revealed that Patient 1 can say 15 words but rarely speaks. She can add and subtract double digits, recognize sight words, and use a few signs. She sometimes becomes agitated and may bite or hit herself or others. Patient 2 can use 50 words, combine 2–3 words, use many signs, write her name, recognize some sight words, and complete some basic addition and subtraction. She occasionally becomes agitated, may hit herself or others, and refuses to cooperate with therapy or schoolwork. Patient 2 receives Ritalin and Clonidine for behavioral problems. A formal neuropsychological assessment was performed at 8 years of age for both sisters and the detailed results are provided in Appendix S1.

**Figure 1:**
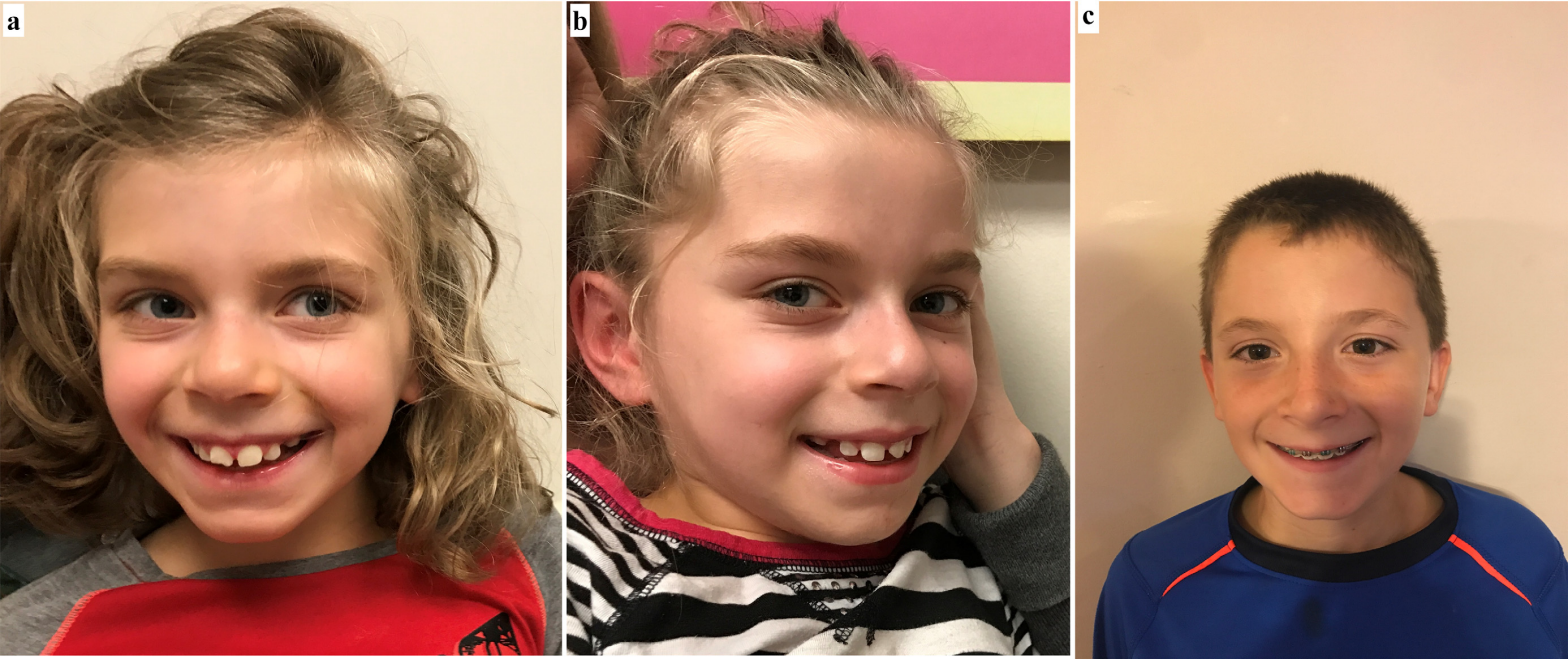
Facial features of Patient 1 & 2 and their brother. Note broad forehead, smooth philtrum, widely spaced teeth and mild retrognathia in proband 1 (a) and proband 2 (b). The brother (c) exhibits no dysmorphic features except for mild hypertelorism

On physical exam at 8 years, Patient 1 and 2 had no major dysmorphic features but were each noted to have a broad forehead, smooth philtrum, widely spaced teeth, high‐arched palate, and mild retrognathia (Figure 1a,b). Their neurological exam revealed mild axial and appendicular hypotonia and drooling. Patient 1's weight was 24 kg (24th centile), height was 126.6 cm (28th centile) and OFC was 53.3 cm (85th centile). Patient 2's weight was 24.7 kg (30th centile), height was 125.4 cm (22nd centile) and OFC was 54.3 cm (95th centile).

The genetic workup of the Patient 1 and 2 started at 3 years of age and included normal karyotype, chromosomal microarray analysis, and fragile X testing. Because of muscle weakness and elevated blood lactate, the two sisters were diagnosed with a nonspecific mitochondrial disease. However, mtDNA sequencing and deletion/duplication studies and a mitochondrial nuclear gene panel were normal except for a variant of unknown significance, p.Asp85Asn, in *KCNE1*. This finding prompted a cardiology consultation and both sisters had a normal echocardiogram and EKG. The exome results (see below) prompted an electroencephalogram which was normal in both sisters.

## 3 MATERIALS AND METHODS

The Institutional Review Board of Washington University School of Medicine approved our study protocol and informed consent was obtained from the parents for publication of genetic results and photographs. Clinical whole‐exome sequencing was performed using a trio design with one of the identical twin sisters submitted as a proband and the second sibling and their parents used for segregation analysis. For more details see Appendix S2.

## 4 MOLECULAR RESULTS

Exome sequencing of the sisters identified a de novo, heterozygous, likely pathogenic variant designated as c.1603A>G (p.Lys535Glu) in *DNM1*. This variant has not been observed in approximately 6,500 individuals that were sequenced as part of the National Heart, Lung, and Blood Institute (NHLBI) exome sequence project or in ExAC database (http://exac.broadinstitute.org/). Both parents tested negative for this variant.

## 5 DISCUSSION

Pathogenic variants in *DNM1* have been reported in association with early‐onset epileptic encephalopathy (including West and Lennox‐Gastaut syndromes), intractable seizures starting within the first year of life, motor impairments, and severe to profound ID (Allen et al., 2016; Deng et al., 2016; Epi4K Consortium; Epilepsy Phenome/Genome Project, 2013; Euro E‐RESC, EpilepsyPhenome/Genome P, Epi4K Constorium, 2014; Nakashima et al., 2016) (Table 1). Other rare clinical features reported in some individuals include hypotonia, developmental regression, movement disorder, autism, cortical visual impairment, behavioral concerns, and microcephaly (Allen et al., 2016; Deng et al., 2016; Epi4K Consortium; Epilepsy Phenome/Genome Project, 2013; Euro E‐RESC, EpilepsyPhenome/Genome P, Epi4K Constorium, 2014; Nakashima et al., 2016) (Table 1). The two patients described here exhibit a milder phenotype with mild to moderate ID, autistic symptoms, and other than a single febrile seizure in Patient 2, no history of seizures.

**Table 1:** Clinical and genetic characteristics of patient with DNM1‐related disorder

Individual (reference)	Case 1 (Nakashima et al., 2016)	Case 2 (Euro E‐RESC, Epilepsy Phenome/Genome P, Epi4K Constorium, 2014)	Case 3 (Deng et al., 2016)	Case 4 (Euro E‐RESC, Epilepsy Phenome/Genome P, Epi4K Constorium, 2014)	Case 5 (Euro E‐RESC, Epilepsy Phenome/Genome P, Epi4K Constorium, 2014)	Case 6 (Euro E‐RESC, Epilepsy Phenome/Genome P, Epi4K Constorium, 2014)	Case 7 (Nakashima et al., 2016)	Case 8 (Allen et al., 2016)	Case 9 (Euro E‐RESC, Epilepsy Phenome/Genome P, Epi4K Constorium, 2014)	Case 10 (Epi4K Consortium; Epilepsy Phenome/Genome Project, 2013)	Proband 1	Proband 2
Domain involved	GTPase	GTPase	GTPase	GTPase	GTPase	GTPase	GTPase	GTPase	Middle	Middle	PH	PH
Mutation	c.127G>A (p.Gly43Ser)	c.194C>A (p.Thr65Asn)	c. 443A>G (p.Glu148Arg)	c.529G>C (p.Ala177Pro)	c.618G>C (p.Lys206Asn)	c.709C>T (p.Arg237Trp)	c.709C>T (p.Arg237Trp)	c.865A>T (p.Ile289Phe)	c.1076G>C (p.Gly359Ala)	c.1190G>A (p.Gly397Asp)	c.1603A>G (p.Lys535Glu)	c.1603A>G (p.Lys535Glu)
Gender, age (years)	M, 15	M, 6	M, 5	F, 15	M, 8	F, 13	M, 6	M, N/A	M, 6	M, N/A	F, 8	F, 8
Diagnosis	LGS	Infantile spasms	Infantile spasms	LGS	Infantile spasms	Infantile spasms	West syndrome	Infantile spasms	LGS	Infantile spasms	Autism, DD	Autism, DD
Development prior to seizure onset	Global DD	Global DD	Some DD	Probably normal	Some DD noted	Global DD	Global DD	Normal	Normal	N/A	N/A	N/A
Seizure onset (months)	11	13	7	7	6	12	10	2	2	Infancy	N/A	N/A
Seizure type at onset	Neck anteflexion, rolling of the eyes and elevating upper limbs	Epileptic spasms	Twitches: Nodded, shrugged, strangled	Epileptic spasms	Epileptic spasms	Epileptic spasms	Atonic type with head dropping	Nodded, slightly ocular myoclonus	Epileptic spasms	N/A	N/A	N/A
Other seizure types/course of seizures	Atonic and tonic seizures, myoclonic seizure	Atypical absences, tonic, focal dyscognitive seizures, obtundation status		Atypical absences with eyelid fluttering, drop attacks, generalized tonic clonic seizures	Atonic and tonic seizures	Myoclonic, atypical absences, focal dyscognitive seizures, generalized tonic clonic seizures, obtundation status	Absences	N/A	Seizure‐free since age 3.5 years	N/A	N/A	Age 5, one episode of unresponsiveness and eye deviation to the left side, characterized by some back arching and leaning over to the left side
EEG at onset of seizures	Partial hypsarrhythmia	Hypsarrhythmia	Sharp wave, slow wave	Slow background, multifocal discharges	Hypsarrhythmia	Modified hypsarrhythmia	Multiple epileptic activity with disorganization of basal activity	Multi – focal epileptic discharge	High voltage bilateral slow spike‐wave discharges	N/A	Normal	Normal
Neurological Findings	N/A	Axial hypotonia	Axial hypotonia	Mild diffuse hypotonia, mild ataxia with wide based gait, mild tremor	General hypotonia	Axial hypotonia, secondary microcephaly	General hypotonia, choric and movements and distal limb dystonia, microcephaly	Deformity, movement disorders	General hypotonia	N/A	Hypotonia	Hypotonia
Brain Imaging/MRI	Normal	Generalized cerebral atrophy	Left ventricle slightly widened	Normal	Normal	Generalized cerebral atrophy	Arachnoid cyst in right temporal lobe	Myelin dysplasia	Normal	N/A	N/A	Normal
Response to treatment/antiepileptic drugs	Intractable, seizure free from 3 to 5 years old with VPA and CLB	Intractable	Intractable, seizure free 4 years old 5 months to 5 years old 5 months age with VPA	Intractable, seizure free from 3 to 8 years of age with VPA and VGB	Intractable, some response to ketogenic diet	Intractable	Intractable	Intractable	Seizure‐free on ketogenic diet	N/A	N/A	N/A
Intellectual Disability	Severe	Profound	Severe	Severe	Severe	Profound	Severe	Profound	Severe	N/A	Mild‐moderate	Mild‐moderate
Development at last follow up	Sit and walk without support; no speech; temporary self‐ harming behavior	No speech; no visual fixation; does not sit or walk	Erect instability, sit alone instability, no language, cannot walk	No speech; autism spectrum disorder; behavioral problems with self‐harm behavior	No speech; does not walk	No speech; no visual fixation; does not sit or walk	Sit and walk without support; no speech	N/A	No speech; does not walk; behavioral problems with self‐ harming behavior	N/A	Sit and walk without support; minimal speech; behavioral problems with self‐harming behavior	Sit and walk without support; limited speech; behavioral problems with self‐harming behavior

CLB, clobazam; DD, developmental delay; F, female; LGS, Lennox Gastaut syndrome; M, male; N/A, not available or applicable; VGB, vigabatrin; VPA, valproic acid.

The twin sisters studied here share the de novo variant, c.1603A>G (p.Lys535Glu) in exon 15 of *DNM1*, classified as likely pathogenic. This variant has not previously been reported in patients or databases of healthy controls. It is a nonconservative amino acid substitution that occurs at an evolutionary conserved position (Figure S1). Previous in vitro studies presented below further support the variant's pathogenicity.

**Figure 2:**
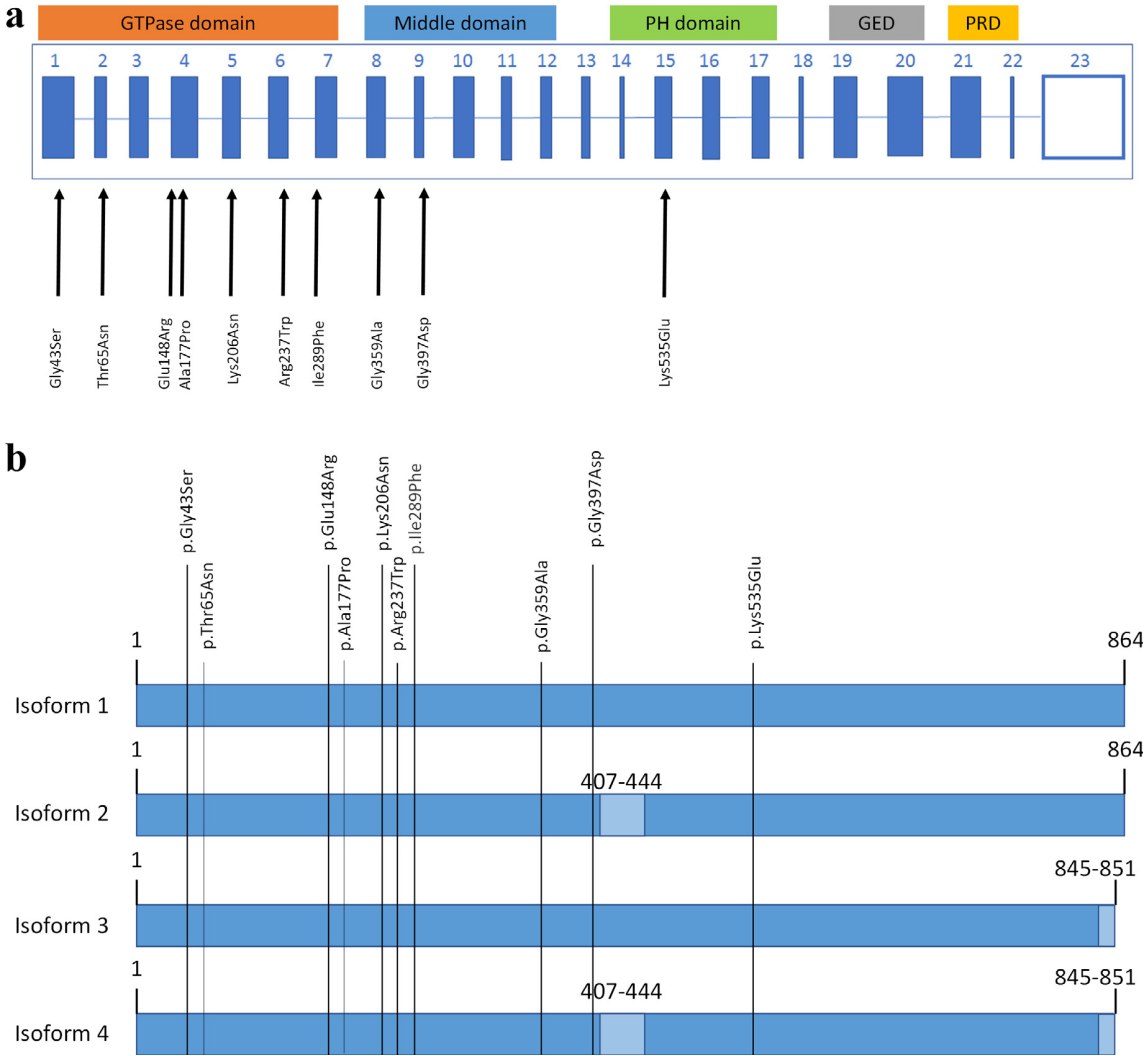
(a) Exons 1–23 of *DNM1*, based on transcript DNM1‐009 (ENST00000341179.11). The genetic variant described in this paper and previously reported pathogenic variants are shown in the diagram. The 5 important Dynamin 1 domains are depicted. (b) Diagram of the four common DNM1 isoforms (RefSeq: NM_004408.3, UniProt: Q05193; dark blue rectangles). Numbers refer to amino acids. The main protein isoform is #1 and has 864 amino acids. Light blue rectangles indicate different amino acids compared to isoform 1. All *DNM1* variants are shown in relation to the different isoforms

Eight previously reported patients with DNM1‐related disorder carry pathogenic variants in the GTPase domain and two carry variants in the middle domain (Figure 2a). To our knowledge, the monozygotic twin sisters in this study have the first reported PH likely pathogenic variant. Our report indicates this variant in the PH domain causes less functional impairments and leads to a less severe phenotype than variants in the GTPase or middle domains.

Dynamin is a family of proteins with GTPase activity that facilitate vesicle scission (Achiriloaie, Barylko, & Albanesi, 1999). In vitro studies showed that the expression of certain GTPase mutants decreased endocytosis activity by interfering with vesicle scission during synaptic vesicle endocytosis and a middle domain mutation disrupted higher order DNM1 oligomerization (Dhindsa et al., 2015). Experiments also showed that when residue 44 in the GTPase domain of *DNM1* was mutated, the protein had reduced binding affinity for GTP and reduced fission activity (Lee, Frank, Marks, & Lemmon, 1999).

The PH domain is primarily responsible for binding PIP2, yet the PH loop has been suggested to have constrictive functioning in the oligomer helix (Achiriloaie, Barylko, & Albanesi, 1999). This structure bends to insert into the plasma membrane and induce curvature before fission. In vitro dominant negative inhibition variants of the PH domain abolish phosphoinositide binding and knock out all synaptic vesicle reuptake (Lee et al., 1999). Variants in the PH domain of dynamin 1 have been previously studied by Vallis et al. (1999) by creating a Lys535Ala mutant. The position Lys535 was chosen because it comes into proximity to the 5'phosphate of the inositol ring in inositol‐1,4,5‐trisphosphate (IP3). The point mutation described in that article is at the same amino acid position as both patients in our study. While Patients 1 and 2 have a Lys535Glu variant, the mutated residue in that study is a Lys535Ala substitution. Each of these amino acid substitutions are significant because they do not share the same structure or chemical properties as lysine. Multiple PH domain mutants were compared, and while others did not show changed levels of transferrin uptake, a well‐established endocytosis assay, Lys535Ala showed inhibition of endocytosis and impairment of PIP2 binding (Vallis et al., 1999). These findings support the critical role of Lys535 in the coordination of phosphoinositide binding, an essential step in endocytosis (Vallis et al., 1999).

A similar study by Achiriloaie et al. (2015) compared PH domain variants K535M and K561M to a GTPase domain variant N272 (missing most of the GTPase domain) and wild‐type *DNM1*. The GTPase domain variant and K535M both inhibited endocytosis, while the K561 mutant and wild type *DNM1* showed no significant difference in endocytosis (Achiriloaie, Barylko, & Albanesi, 1999). This implies that not all amino acid substitutions in the PH domain are significant and reinforces the claim that Lys535 is essential to the PH loop binding to PIP2 and promoting endocytosis. PH domain mutations at other sites may not have a clinically discernable phenotype. We also examined whether the position of the pathogenic variants affects different isoforms of Dynamin 1, but found all variants involve all isoforms (Figure 2b).

In summary, Dynamin 1 is mainly expressed in the central nervous system, explaining the neurological phenotype in *DNM1*‐related disorder. Previously reported patients carry pathogenic variants in the GTPase or middle domains and presented with epileptic encephalopathy and severe neurodevelopmental symptoms. Here, we report the first documented *DNM1* variant in the PH domain associated with a milder phenotype without epilepsy. Our data suggest a novel, possibly unique, and less severe phenotype resulting from a variant in the PH domain of dynamin 1. Reports of additional patients are needed to better define the genotype–phenotype correlation.

## CONFLICTS OF INTEREST

AT discloses her employment with GeneDx. EB, EF, GCA, JD, SJP, and MS declare no conflicts of interest.

## References

[mgg3362-bib-0001] Achiriloaie, M. , Barylko, B. , & Albanesi, J. P. (1999). Essential role of the dynamin pleckstrin homology domain in receptor‐mediated endocytosis. Molecular and Cellular Biology, 19(2), 1410–1415. https://doi.org/10.1128/MCB.19.2.1410 9891074 10.1128/mcb.19.2.1410 PMC116069

[mgg3362-bib-0002] Allen, N. M. , Conroy, J. , Shahwan, A. , Lynch, B. , Correa, R. G. , Pena, S. D. , … King, M. D. (2016). Unexplained early onset epileptic encephalopathy: Exome screening and phenotype expansion. Epilepsia, 57(1), e12–e17. https://doi.org/10.1111/epi.13250 26648591 10.1111/epi.13250

[mgg3362-bib-0003] Antonny, B. , Burd, C. , De Camilli, P. , Chen, E. , Daumke, O. , Faelber, K. , … Kirchhausen, T. (2016). Membrane fission by dynamin: What we know and what we need to know. EMBO Journal, 35(21), 2270–2284. https://doi.org/10.15252/embj.201694613 27670760 10.15252/embj.201694613 PMC5090216

[mgg3362-bib-0004] Deng, X. L. , Yin, F. , Zhang, C. L. , Ma, Y. P. , He, F. , Wu, L. W. , & Peng, J. (2016). Dynamin‐1‐related infantile spasms: A case report and review of literature. Zhonghua Er Ke Za Zhi, 54(11), 856–859. 27806796 10.3760/cma.j.issn.0578-1310.2016.11.014

[mgg3362-bib-0005] Dhindsa, R. S. , Bradrick, S. S. , Yao, X. , Heinzen, E. L. , Petrovski, S. , Krueger, B. J. , … Goldstein, D. B. (2015). Epileptic encephalopathy‐causing mutations in DNM1 impair synaptic vesicle endocytosis. Neurology Genetics, 1(1), e4 https://doi.org/10.1212/01.NXG.0000464295.65736.da 27066543 10.1212/01.NXG.0000464295.65736.da PMC4821085

[mgg3362-bib-0006] Epi4K Consortium; Epilepsy Phenome/Genome Project , Allen, A. S. , Berkovic, S. F. , Cossette, P. , Delanty, N. , Dlugos, D. , … Winawer, M. R. (2013). De novo mutations in epileptic encephalopathies. Nature, 501:217–221 https://doi.org/10.1038/nature12439 23934111 10.1038/nature12439 PMC3773011

[mgg3362-bib-0007] Euro E‐RESC, EpilepsyPhenome/Genome P, Epi4K Constorium (2014). De novo mutations in synaptic transmission genes including DNM1 cause epileptic encephalopathies. American Journal of Human Genetics, 95:360–370. https://doi.org/10.1016/j.ajhg.2014.08.013 25262651 10.1016/j.ajhg.2014.08.013 PMC4185114

[mgg3362-bib-0008] Lee, A. , Frank, D. W. , Marks, M. S. , & Lemmon, M. A. (1999). Dominant‐negative inhibition of receptor‐mediated endocytosis by a dynamin‐1 mutant with a defective pleckstrin homology domain. Current Biology, 9(5), 261–264. https://doi.org/10.1016/S0960-9822(99)80115-8 10074457 10.1016/s0960-9822(99)80115-8

[mgg3362-bib-0009] McNiven, M. (2000). The dynamin family of mechanoenzymes: Pinching in new places. Trends in Biochemical Sciences, 25(3), 115–120. https://doi.org/10.1016/S0968-0004(99)01538-8 10694881 10.1016/s0968-0004(99)01538-8

[mgg3362-bib-0010] Nakashima, M. , Kouga, T. , Lourenço, C. M. , Shiina, M. , Goto, T. , Tsurusaki, Y. , … Osaka, H. (2016). De novo *DNM1* mutations in two cases of epileptic encephalopathy. Epilepsia, 57(1), e18–e23. https://doi.org/10.1111/epi.13257 26611353 10.1111/epi.13257

[mgg3362-bib-0011] Rizzoli, S. O. (2014). Synaptic vesicle recycling: Steps and principles. EMBO Journal, 33(8), 788–822. https://doi.org/10.1002/embj.201386357 24596248 10.1002/embj.201386357 PMC4194108

[mgg3362-bib-0012] Romeu, A. , & Arola, L. (2014). Classical dynamin *DNM1* and DNM3 genes attain maximum expression in the normal human central nervous system. BMC Research Notes, 28(7), 188 https://doi.org/10.1186/1756-0500-7-188 10.1186/1756-0500-7-188 PMC3986822 24673776

[mgg3362-bib-0013] Sudhof, T. C. (2004). The synaptic vesicle cycle. Annual Review of Neuroscience, 27, 509–547. https://doi.org/10.1146/annurev.neuro.26.041002.131412 10.1146/annurev.neuro.26.041002.131412 15217342

[mgg3362-bib-0014] Takei, K. (1996). The synaptic vesicle cycle: A single vesicle budding step involving clathrin and dynamin. Journal of Cell Biology, 133(6), 1237–1250. https://doi.org/10.1083/jcb.133.6.1237 8682861 10.1083/jcb.133.6.1237 PMC2120898

[mgg3362-bib-0015] Vallis, Y. , Wigge, P. , Marks, B. , Evans, P. R. , McMahon, H. T. (1999). Importance of the pleckstrin homology domain of dynamin in clathrin‐mediated endocytosis. Current Biology, 9(5), 257–260. https://doi.org/10.1016/S0960-9822(99)80114-6 10074456 10.1016/s0960-9822(99)80114-6

