# Supplementary material for: Mutations in the PH Domain of DNM1 are associated with a nonepileptic phenotype characterized by developmental delay and neurobehavioral abnormalities
Source: Mol Genet Genomic Med. 2018 Feb 4;6(2):294–300. doi: 10.1002/mgg3.362 (PMC5902389; doi:10.1002/mgg3.362)
Supplement: Supplementary file 1 [file MGG3-6-294-s001.docx]

**Supplementary Material**

**Appendix S1 :Clinical Report**

**Neuropsychological Assessment of the Patients at Age 8 years:**

Patient 1 presented to neuropsychological testing as a compliant but detached youngster. She was heard to imitate the examiner’s word “mouth” on one occasion, but otherwise was nonverbal and did not communicate using spoken language. She was also somewhat socially detached and exhibited little emotional expression during the evaluation. On formal testing, the patient’s overall intellectual ability as assessed using a language-free measure fell far below average. Within that context, her neuropsychological profile highlighted widespread deficits encompassing receptive vocabulary, visual-motor skills, attention, and executive functions. Pre-academic concept knowledge and parent ratings of the patient’s adaptive behavior also fell far below average. Diagnostically, the patient’s presentation continued to be consistent with her previously identified autism spectrum disorder. Her intellectual ability and adaptive functioning also fell sufficiently below average to be consistent with mild to moderate intellectual disability, in conjunction with autism spectrum disorder.

Patient 2 was initially shy and anxious, and had difficulty separating from her mother to begin testing. She eventually was able to begin testing unaccompanied by her mother using a blanket and books as transitional objects. The patient did not communicate with the examiner for the majority of the evaluation and was somewhat difficult to engage socially. However, she used American Sign Language (ASL) to provide “yes” or “no” responses to verbal test questions. The patient was not overtly defiant or disruptive, but was very inattentive and easily distracted during testing. She responded well to using her blanket, books, snacks, and stickers as reinforcement to ensure her attention to tasks. On formal testing, her overall intellectual ability as assessed using a language-free measure was far below average. Additional testing documented widespread deficits encompassing her receptive vocabulary, visual-motor skills, and attention. Parent ratings of the patient’s adaptive behavior also fell far below average. Diagnostically, the patient’s intellectual ability and adaptive functioning fell sufficiency below average to be consistent with moderate intellectual disability. She also was determined to meet criteria for separation anxiety disorder. Additionally, her presentation continued to be consistent with her previously identified autism spectrum disorder, and she continued to display marked deficits in attention beyond that which would be expected for her mental age, consistent with her previously diagnosed ADHD.

| **Standardized neuropsychological test scores for patients 1 and 2** | | |
| --- | --- | --- |
| **Domain** | **Patient 1** | **Patient 2** |
| Intellectual Ability |  |  |
| Nonverbal IQ | 62 | 60 |
| Verbal Skills |  |  |
| Receptive Vocabulary | 23 | 28 |
| Visual-Motor Skills |  |  |
| Visual-Motor Integration | <45 | <45 |
| Adaptive Behavior |  |  |
| General Adaptive Composite | 52 | 53 |

Note. Standard scores have a mean of 100 and a SD of 15.

**Appendix S2 Materials and Methods**

After informed consent was obtained for clinical whole exome sequencing, DNA was isolated from the siblings and their parents using standard methods. Whole exome sequencing was performed using a trio design with one of the identical twin sisters submitted as a proband and the second sibling and biological parents used for segregation analysis. Exon targets were isolated by capture using the Agilent Clinical Research Exome kit (Agilent Technologies, Santa Clara, CA) and sequenced on the Illumina HiSeq 2000 system with 100 bp paired-end reads. Sequence data were assembled using human genome build GRCh37/UCSC hg19. The mean depth of coverage was 159x, with ≥10x coverage across 97.5% of the exome. Variants were evaluated using Xome Analyzer software (GeneDx, Gaithersburg, MD). Zygosity testing for the siblings was performed by comparing the genotyping results in a standard panel of 15 highly polymorphic loci using a short tandem repeat multiplex assay (AmpFIRST Identifiler Direct PCR Amplification kit). Kinship coefficients from exome data were calculated using kinship-based inference to control for misspecified relationships and unreported consanguinity for relations as distant as second cousins. Capillary sequencing confirmed the variant p.Lys535Glu (AAA>GAA): c.1603A>G in exon 15 in *DNM1* (NM_004408.3).


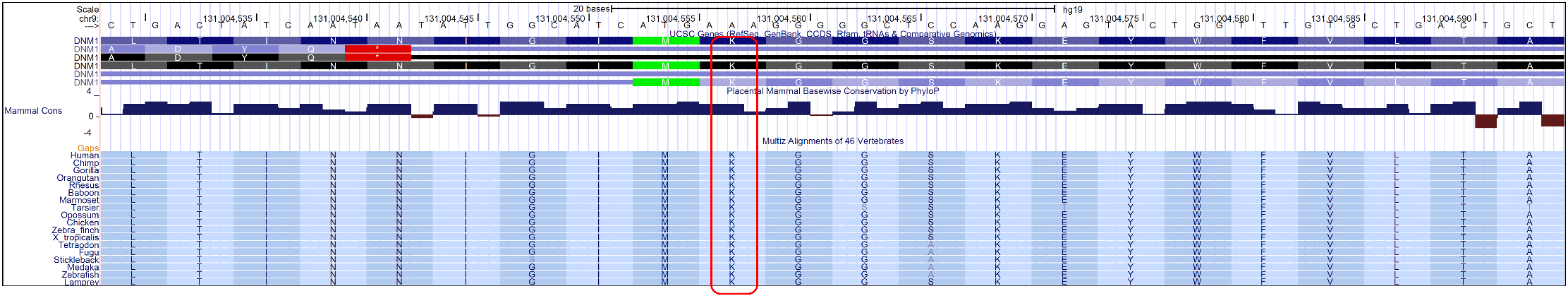


**Figure S1**. A track shows multiple alignments of vertebrate species for dynamin 1 generated by using multiz and other tools in the UCSC (<https://genome.ucsc.edu> ) /Penn State Bioinformatics comparative genomics alignment pipeline. The evolutionary conserved amino acid lysine (K) at position 535 is shown in red rectangle
